# A comparative study of SNPscan/CNVplex assay and routine PCR in genetic analysis of thalassemia

**DOI:** 10.3389/fgene.2026.1819795

**Published:** 2026-06-26

**Authors:** Xiufen Bu, Yang Sun, Siyi Ding, Can Peng, Mengyue Yang, Guo Zeng, Shihao Zhou, Siyuan Linpeng, Li Zeng, Jing Liu

**Affiliations:** Hunan Provincial Key Laboratory of Regional Hereditary Birth Defects Prevention and Control, Changsha Hospital for Maternal and Child Health Care Affiliated to Hunan Normal University, Changsha, China

**Keywords:** CNVplex, high throughput, molecular diagnosis, SNPscan, thalassemia, triplications

## Abstract

**Background:**

Accurate molecular diagnosis is essential for thalassemia prevention and carrier screening, particularly in high-prevalence regions. Conventional PCR-based methods are widely used in clinical practice but have limited ability to detect rare variants, homologous recombination events, and large structural rearrangements. This study evaluated the clinical performance of combined SNPscan/CNVplex assay for comprehensive thalassemia screening.

**Methods:**

A total of 1,026 hematologic testing-positive individuals from Hunan Province, China, were analyzed using routine PCR and SNPscan/CNVplex assay in parallel. The SNPscan assay targeted 48 single-nucleotide variants and indels, while CNVplex targeted 28 deletional mutations and homologous recombination events in *HBA1*/*HBA2* and *HBB*. All positive results and discordant results were confirmed by Sanger sequencing or specially designed PCR.

**Results:**

SNPscan/CNVplex assay identified thalassemia-associated variants in 302 individuals (29.43%), compared with 283 correctly detected by routine PCR, representing a 6.71% increase in diagnostic yield. Concordant results between the two methods were observed in 98.15% of cases, while 19 cases showed discordant findings. Additional variants detected by SNPscan/CNVplex assay included 14 α-globin gene triplications, one HKαα rearrangement, two rare SNVs/indels, and two large fragment copy number variants involving the α-globin gene cluster. The most common α-thalassemia mutation was--^SEA^, whereas *HBB*:c.316-197C>T was the most frequent β-thalassemia variant. Three individuals carried both α-globin gene triplications and β-thalassemia mutations, indicating potential risk for aggravated globin chain imbalance. CNVplex assay also successfully identified large deletions and duplications encompassing the α-globin gene cluster that were missed by routine PCR.

**Conclusion:**

Combined SNPscan/CNVplex assay demonstrated broader mutation coverage and higher diagnostic yield than routine PCR, particularly for detecting homologous recombination events and large structural variants. This integrated strategy may improve carrier detection and genetic risk assessment in thalassemia screening programs.

## 1 Introduction

Thalassemia is a common monogenic inherited hemoglobinopathy caused by impaired synthesis of either α- or β-globin chains (Kattamis et al., 2022). It is primarily prevalent in tropical and subtropical regions (Weatherall, 2018). Globally, approximately 5% of the population carries α-thalassemia mutations, while 1.5% carries β-thalassemia mutations (Gao and Liu, 2022). Heterozygous carriers exhibit a certain survival advantage in malaria-endemic areas, which contributes to the high prevalence of thalassemia in regions such as the Mediterranean, the Middle East, South Asia, Southeast Asia, and Africa (Piel and Weatherall, 2014). ⍺-Thalassemia is primarily characterized by large-scale deletions or, less frequently, point mutations within the *HBA1* and *HBA2* genes on chromosome 16p13.3, with clinical severity being directly proportional to the number of inactivated ⍺-globin alleles (Piel and Weatherall, 2014). Single or double-allele inactivation typically presents as asymptomatic or mild carrier states, whereas triple-allele deletions lead to Hemoglobin H (HbH) disease, and the complete loss of all four alleles results in the fatal Hb Bart’s hydrops fetalis syndrome (Leung and Lao, 2012). While genomic deletions mediated by homologous recombination are the primary drivers, non-deletional mutations often contribute to disproportionately severe clinical manifestations through compromised mRNA stability or translation (Kalle Kwaifa et al., 2020). Parallel to this, β-thalassemia is caused by mutations within the *HBB* gene, ranging from point mutations to small indels, which quantitatively reduce or abolish β-globin production (Taher et al., 2021). The phenotypic severity is strictly genotype-dependent, where β^0^/β^0^ or β^+^/β^0^ genotypes manifest as severe, transfusion-dependent anemia early in life (Wood, 2023). The epidemiological landscape of thalassemia in China is marked by a substantial carrier population of approximately 30 million, primarily localized within the southern coastal regions. This high carrier frequency translates into one of the most significant global incidences of severe thalassemia phenotypes (Huang et al., 2019). Despite the success of prenatal screening in high-income countries, a great number of new cases continue to emerge annually in China (Wang et al., 2023). Severe cases necessitate lifelong transfusion dependence and iron chelation (Viprakasit et al., 2014), which bring substantial direct and indirect costs. Furthermore, long-term complications such as cardiac and hepatic iron toxicity, endocrine dysfunction, and growth failure, significantly affect patient quality of life (Leung and Lao, 2012). Therefore, it remains imperative for the multi-tiered healthcare system to strengthen prevention strategies including expended genetic screening, evidence-based prenatal interventions and effective long-term management protocols.

It recommends preconception or early pregnancy screening with complete blood count, iron studies, and hemoglobin electrophoresis (ACOG Committee on Obstetrics, 2007). Given the limited sensitivity of hematologic parameters in some carriers, especially in high-risk groups, molecular genetic testing is considered the gold standard for accurate diagnosis (Fucharoen and Weatherall, 2016). Genotyping not only confirms carrier status but also guides genetic counseling and reproductive planning. Currently, for non-deletional mutations, Sanger sequencing remains the gold standard for the identification of both known and novel point mutations, due to its direct and accurate DNA sequencing capability (Tan et al., 1992). For known hotspot mutations, targeted methods such as reverse dot blot hybridization (RDB), amplification refractory mutation system PCR (ARMS-PCR), and restriction fragment length polymorphism (RFLP) are commonly employed (Shah et al., 2020; Zhong et al., 2023). In the detection of deletional mutations, gap-PCR is suitable for identifying large deletions with defined breakpoints. Meanwhile, multiplex ligation-dependent probe amplification (MLPA) can simultaneously detect both known and unknown deletions or duplications, although it lacks the ability to precisely define breakpoint locations (Tang et al., 2023). In China, the 23 to 30 most common variants in *HBA* and *HBB* genes are routinely screened by those traditional methods in diagnostic laboratories of thalassemia. While these methods offer high cost-effectiveness and clinical longevity, they are limited to high-frequency hotspot mutations, have low throughput, and involve subjectivity in thalassemia screening results. With the advancement of high-throughput sequencing technologies, both next-generation sequencing (NGS) and third-generation sequencing (TGS) have been increasingly applied in the molecular classification and diagnosis of thalassemia (Xu et al., 2023). NGS enables parallel sequencing of short DNA fragments, while TGS offers long-read capabilities and superior performance in GC-rich regions, making it advantageous for the detection of complex structural variants, greatly improving detection rates (Huang et al., 2024). Nevertheless, the widespread application of these techniques in primary healthcare settings remains limited due to their relatively high costs and complex data analysis requirements.

CNVplex is a patented copy number variations (CNVs) detection technology based on capillary electrophoresis that combines multiplex amplification with probe hybridization for quantitative analysis of multiple targets in a single reaction (Xu et al., 2025). When combined with the multiplex single nucleotide polymorphism (SNP) genotyping platform SNPscan, it allows simultaneous detection of common CNVs and SNP variants in specific genes (Wei et al., 2024). The advantages of SNPscan and CNVplex assay were that: first, the two technologies are based on the principle of high specificity connection, with simple operation, high sensitivity and accuracy; second, the assay system can simultaneously detect multiple types of mutations by designing probes/primers of different lengths and fluorescence. This integrated and efficient approach shows promise as a screening tool for genetic risk assessment of thalassemia. In this study, we applied a thalassemia carrier screening system developed based on the combined CNVplex and SNPscan technologies to 1,026 archived samples from Hunan Province, China. The results were compared with previously established routine PCR data to evaluate the clinical utility and potential of CNVplex assay in combination with SNPscan assay for thalassemia risk prevention and control.

## 2 Materials and methods

### 2.1 Subjects

**FIGURE 1:**
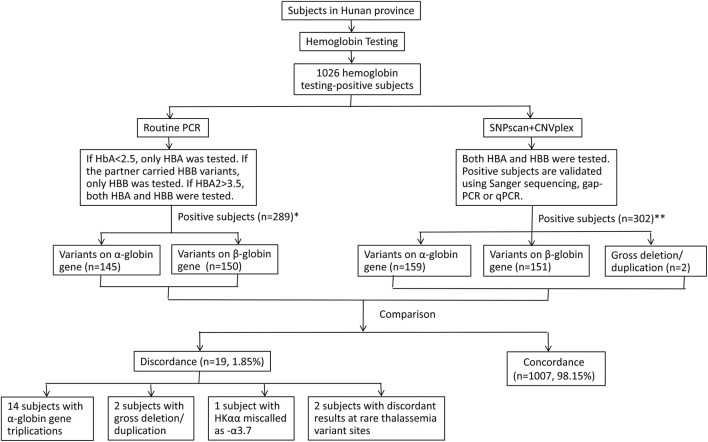
Flowchart of the study. PCR, polymerase chain reaction; *, six cases carried two variants; **, ten cases carried two variants.

The study design and protocol were reviewed and approved by the ethics committee of *Changsha Hospital for Maternal and Child Health Care*. All methods and clinical procedures were performed in accordance with the relevant guidelines and regulations. All participants received genetic counseling and provided informed consent before testing. Hematologic testing was conducted for these subjects. From January to December 2025, there were 1,026 consecutive series of hematologic testing-positive samples, comprising 108 males and 918 females aged from 1 month to 52 years. SNPscan/CNVplex assay and routine PCR were simultaneously performed for 1,026 hematologic testing-positive subjects (Figure 1).

### 2.2 Hematologic testing

Hematologic testing was carried out by standard blood assays and Hb electrophoresis. Standard blood assays were conducted with an automated cell counter (Sysmex XN-1000, Sysmex Co., Ltd., Kobe, Japan). Blood indexes included mean corpuscular volume, mean corpuscular Hb, and Hb. An Hb electrophoresis system (Capillarys 2 Flex Piercing, Sebia, Evry Cedex, France) was used to analyze the Hb components, which included HbA_2_ and HbF. Normal ranges were mean corpuscular volume (MCV) 80 fL or higher, mean corpuscular hemoglobin (MCH) 27 pg or higher, HbA_2_ levels between 2.5% and 3.5%, and HbF 5% or lower.

### 2.3 Genetic analysis by routine PCR

Routine PCR was carried out to test α-thalassemia variants including--^SEA^ (Southeast Asia), -α^3.7^ (rightward), -α^4.2^ (leftward), *HBA2*:c.427T>C, *HBA2*:c.377T>C, and *HBA2*:c.369C>G and β-thalassemia variants including *HBB*:c.126_129delCTTT, *HBB*:c.130G>T, *HBB*:c.316-197C>T, *HBB*:c.52A>T, *HBB*:c.45-46insG, *HBB*:c.-76A>G, *HBB*:c.-79A>G, *HBB*:c.216_217insA, *HBB*:c.79G>A, *HBB*:c.92+1G>T, *HBB*:c.92+1G>A, *HBB*:c.84_85insC, *HBB*:c.92+5G>C, *HBB*:c.-11_-8delAAAC, *HBB*:c.-50A>C, *HBB*:c.2T>G, *HBB*:c.94delC, *HBB*:c.-80T>C, and *HBB*:c.-82C>A. The experiment was performed according to the manufacturer’s protocol (Kaipu Bioscience, Chaozhou, China).

### 2.4 SNPscan assay

The main processes of SNPscan assay include design probes for each locus, ligation reaction, PCR amplification of linked products, genotype analysis (Flowchart of SNPscan show in Supplementary Figure S1). The experimental protocol was carried out in the following manner: DNA samples were diluted to a concentration ranging from 30 to 50 ng/μL. A 4 μL sample was mixed with 2.5 μL of 4×DNA lysis buffer and the volume was adjusted to 10 μL with nuclease-free water. The mix was incubated at 98 °C for 5 min then immediately place on ice. Added 10 μL of ligation premix (comprising 2 μL of 10×ligation buffer, 0.5 μL of ligase, 1 μL of probe mix and 6.5 μL of ddH2O) and place in a Thermal Cycler (e.g., ABI 2720) to initiate the program: 4x (94 °C 1min, 58 °C 4h), 94 °C 2 min, a final hold at 72 °C. Following ligation, 20 μL of EDTA (20 mM) was added to stop the reaction. Prepare multiplex fluorescent PCR amplification mix consisting of 10 μL of 2 × PCR Master Mix, 1 μL of primer mix, 1 μL of ligation product and 8 μL of ddH2O.Then run the following thermal cycling program:95 °C for 2 min, 9x (94 °C 20 s, 62 °C–0.5 °C/cycle 40 s, 72 °C 1.5 min) 25x (94 °C 20 s, 57 °C 40 s, 72 °C 1.5 min),followed by 60 °C for 1h and a 4 °C hold. The PCR products were diluted 10-fold. An aliquot of 1 μL was mixed with 0.5 μL of Liz500 Size Standard and 8.5 μL of Hi-Di formamide. The mixture was denatured at 95 °C for 5 min and then analyzed on an ABI 3730XL Genetic Analyzer. A panel of 48 variants in *HBA1/HBA2* and *HBB* genes were designed by Genesky Diagnostics (Suzhou) Inc. for detected 1,026 samples (Table 1).

**TABLE 1:** 48 variants in HBA1/HBA2 and HBB.

NO.	Gene	Transcript	Mutation
1	*HBA1*	NM_000558.5	c.223G>C
2	*HBA1*	NM_000558.5	c.358C>T
3	*HBA2*	NM_000517.6	c.*92A>G
4	*HBA2*	NM_000517.6	c.40G>T
5	*HBA2*	NM_000517.6	c.91_93delGAG
6	*HBA2*	NM_000517.6	c.95G>A
7	*HBA2*	NM_000517.6	c.99G>A
8	*HBA2*	NM_000517.6	c.358C>T
9	*HBA2*	NM_000517.6	c.369C>G
10	*HBA2*	NM_000517.6	c.377T>C
11	*HBA2*	NM_000517.6	c.427T>C
12	*HBB*	NM_000518.5	c.-140C>T
13	*HBB*	NM_000518.5	c.-100G>A
14	*HBB*	NM_000518.5	c.-82C>A
15	*HBB*	NM_000518.5	c.-81A>C
16	*HBB*	NM_000518.5	c.-81A>G
17	*HBB*	NM_000518.5	c.-80T>C
18	*HBB*	NM_000518.5	c.-79A>G
19	*HBB*	NM_000518.5	c.-78A>C
20	*HBB*	NM_000518.5	c.-78A>G
21	*HBB*	NM_000518.5	c.-50A>C
22	*HBB*	NM_000518.5	c.-10_-7delAAAC
23	*HBB*	NM_000518.5	c.2T>G
24	*HBB*	NM_000518.5	c.17_18delCT
25	*HBB*	NM_000518.5	c.25_26delAA
26	*HBB*	NM_000518.5	c.27dupG
27	*HBB*	NM_000518.5	c.45dupG
28	*HBB*	NM_000518.5	c.52A>T
29	*HBB*	NM_000518.5	c.79G>A
30	*HBB*	NM_000518.5	c.85dupC
31	*HBB*	NM_000518.5	c.91A>G
32	*HBB*	NM_000518.5	c.92+1G>T
33	*HBB*	NM_000518.5	c.92+2T>C
34	*HBB*	NM_000518.5	c.92+5G>C
35	*HBB*	NM_000518.5	c.92+6T>C
36	*HBB*	NM_000518.5	c.93-21G>A
37	*HBB*	NM_000518.5	c.94delC
38	*HBB*	NM_000518.5	c.113G>A
39	*HBB*	NM_000518.5	c.126_129delCTTT
40	*HBB*	NM_000518.5	c.130G>T
41	*HBB*	NM_000518.5	c.162delT
42	*HBB*	NM_000518.5	c.165_177delTATGGGCAACCCT
43	*HBB*	NM_000518.5	c.216dupT
44	*HBB*	NM_000518.5	c.217dupA
45	*HBB*	NM_000518.5	c.315+1G>A
46	*HBB*	NM_000518.5	c.315+5G>C
47	*HBB*	NM_000518.5	c.316-197C>T
48	*HBB*	NM_000518.5	c.383_385delAGG

### 2.5 CNVplex assay

The experimental protocol for the CNVplex Assay was identical to that of the SNPscan assay (Flowchart of CNVplex show in Supplementary Figure S2). GeneMapper v5.0 software was used to collect raw data and export peak height data of PCR amplification product peaks at each locus. Output the peak heights value (H value) from each locus. The ratio of the target peak height value to the reference peak height value within the same group was calculated to obtain the R value, while the corresponding ratio for control samples was designated as R’. The copy number of the target region was determined by multiplying the R/R′ ratio by the copy number of the control sample (2 for diploids). Based on the rounding principle, the copy number of the target region were assigned as follows:0 if it is less than 0.5, 1 if 0.5 ≤ values<1.5, 2 if 1.5 ≤ values<2.5, and so on. For quality control (QC), copy number detection values for each locus were assigned level of 1 if they fell within the ranges of 0.00–0.10, 0.80–1.20, 1.70–2.30, 2.70–3.30, and 3.60–4.40. The proportion of probes achieving this quality level was used as a critical parameter for evaluating overall data quality. Samples with a proportion exceeding 80% were considered to have passed QC and were subsequently included in the final copy number analysis. To distinguish genuine CNVs from data fluctuations, we applied the following criteria: a CNV was defined as a consistent change across two or more consecutive sites, whereas an alteration at a single primer site was considered a data fluctuation. A panel of 28 deletional mutations and homologous recombination in *HBA1/HBA2* and *HBB* genes were designed by Genesky Diagnostics (Suzhou) Inc. for detected 1,026 samples in this study (Table 2).

**TABLE 2:** 28 deletional mutations and homologous recombination in *HBA1*/*HBA2* and *HBB*.

NO.	Gene	Mutation
1	*HBA*	--^SEA^
2	*HBA*	-α^3.7^
3	*HBA*	-α^4.2^
4	*HBA*	-α^2.4^
5	*HBA*	-α^27.6^
6	*HBA*	--^THAI^
7	*HBA*	--^FIL^
8	*HBA*	--^MED^
9	*HBA*	--^20.5^
10	*HBA*	HS-40
11	*HBA*	-α^21.9^
12	*HBA*	-α^MAL3.5^
13	*HBA*	-α^2.8^
14	*HBA*	--^11.1^
15	*HBA*	--^9.7^
16	*HBA*	Other large fragment deletion mutations
17	*HBA*	ααα^anti3.7^
18	*HBA*	ααα^anti4.2^
19	*HBA*	HKαα
20	*HBA*	ααα^antiHKαα^
21	*HBB*	Chinese (Aγδβ)0
22	*HBB*	HPFH-6
23	*HBB*	HPFH-S.E.Asian
24	*HBB*	Thai (δβ)0-Thal
25	*HBB*	Filipino
26	*HBB*	Taiwanese
27	*HBB*	Lepore-Boston-Washington
28	*HBB*	Other large fragment deletion mutations

### 2.6 Confirmation of discordant variants

All positive results from the SNPscan/CNVplex assay have been validated by Sanger sequencing or specially designed PCR. Furthermore, discordant structural variants identified by SNPscan/CNVplex assay but missed by routine PCR were verified by gap-PCR or qPCR. Discordant SNVs and indels between SNPscan/CNVplex assay and routine PCR were validated by Sanger sequencing.

## 3 Results

### 3.1 Comparison between SNPscan/CNVplex assay and routine PCR

SNPscan/CNVplex assay and routine PCR were conducted for 1,026 hemoglobin testing-positive subjects side by side (Figure 1). Of these subjects, 1,007 (98.15%) had the same results whereas 19 (1.85%) exhibited discordant results between the two methods. Among the 19 discordant results, one variant was misidentified by routine PCR, and 18 were beyond the PCR’s predefined detection range. Specifically, these 18 variants comprised 14 α-globin gene triplication, two rare SNVs/indels, and two gross deletions/duplications. In total, SNPscan/CNVplex assay correctly identified variants in 302 subjects compared to 283 identified by routine PCR, representing a 6.71% increase in the diagnostic yield.

In 1,026 subjects with hemoglobin testing–positive, we detected a total of 26 types of mutations in *HBA1/HBA2* or *HBB* in 302 cases by SNPscan/CNVplex assay, with a positivity rate of 29.43% (302/1,026) (Table 3). *HBA1/HBA2* mutations were identified in 159 samples, the most common variant was *HBA*: -^SEA^ (n = 111). We also identified *HBB* mutations in 151 samples; different from *HBA* mutations, all *HBB* mutations were non-large-fragment deletion mutations. The *HBB*:c.316-197C>T mutation (n = 50) was the most common in our β-thalassemia samples. The results of genetic screening in these 1,026 Chinese individuals are shown in Supplementary Table S1.

**TABLE 3:** Distribution of α- and β-thalassemia genotypes by analysis of thalassemia alleles.

Genotype	Detected on SNPscan/CNVplex assay	
	Samples (n)	Frequency (%)
Single α-globin gene deletion		
-α^3.7^/αα	3	0.96%
-α^4.2^/αα	13	4.17%
--^SEA^/ααα^anti4.2^	1	0.32%
Two α-globin gene deletions		
--^SEA^/αα	111	35.58%
-α^3.7^/-α^3.7^	1	0.32%
-α^4.2^/-α^4.2^	1	0.32%
Three α-globin gene deletions		
--^SEA^/-α^4.2^	1	0.32%
α-globin gene triplication		
ααα^anti3.7^/αα	10	3.21%
ααα^anti4.2^/αα	3	0.96%
Unusual a-globin arrangements		
HKαα	1	0.32%
α-globin gene point mutation		
*HBA2*: c.427T>C Hete	8	2.56%
*HBA2*: c.377T>C Hete	4	1.28%
*HBA2*: c.369C>G Hete	1	0.32%
*HBA1*: c.223G>C Hete	1	0.32%
β-globin gene point mutation		
*HBB*: c.316-197C>T Hete	50	16.03%
*HBB*: c.126_129delCTTT Hete	44	14.10%
*HBB*: c.52A>T Hete	27	8.65%
*HBB*: c.217dupA Hete	8	2.56%
*HBB*: c.-78A>G Hete	8	2.56%
*HBB*: c.79G>A Hete	6	1.92%
*HBB*: c.45dupG Hete	3	0.96%
*HBB*: c.85dupC Hete	3	0.96%
*HBB*: c.130G>T Hete	1	0.32%
*HBB*: c.-10_-7delAAAC Hete	1	0.32%
Gross deletion/duplication	2	0.64%
Total	302*	100%

* Ten cases carried two variants.

### 3.2 Additional thalassemia variants detected by SNPscan/CNVplex assay

**FIGURE 2:**
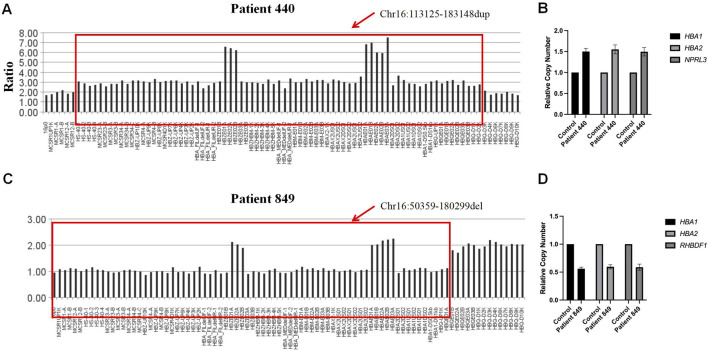
Large fragment deletion or duplication of Patient 440 and 849. **(A)** CNVplex analysis of the position of α-globin gene clusters deletion coordinates in Patient 440. **(B)** Results of qPCR in Patient 440, who has the duplication of *HBA1*, *HBA2*, and *NPRL3*. **(C)** CNVplex analysis of the position of α-globin gene clusters deletion coordinates in Patient 849. **(D)** Results of qPCR in Patient 849, who has the deletion of *HBA1*, *HBA2*, and *RHBD1*.

In addition to the thalassemia variants tested by routine PCR, SNPscan/CNVplex assay can identify some rare variants in α and β-globin genes. As mentioned above, 302 samples carrying *HBA1/HBA2* or *HBB* mutations were detected by the SNPscan and CNVplex assay. And in 19 of them, mutations were not identified or misdiagnosed by the routine PCR (Table 4). As shown in Table 4, 10 samples were detected with *HBA*:ααα^anti3.7^/αα and 4 samples with *HBA2*:ααα^anti4.2^/αα by the SNPscan/CNVplex assay. Notably, two samples had the genotype *HBA*:ααα^anti3.7^/αα with a heterozygous *HBB*:c.126_129delCTTT mutation (Patient 369 and 739), and one had the genotype *HBA2*:ααα^anti4.2^/αα with a heterozygous *HBB*:c.316-197C>T mutation (Patient 190). These three cases represent a complex molecular profile characterized by the simultaneous presence of β-thalassemia mutations and α-globin gene triplication. In a notable instance, Sample 312 was identified as having the HKαα/αα genotype by the SNPscan/CNVplex assay, whereas the routine PCR identified it as -α^3.7^/αα. Subsequent sanger sequencing validated the SNPscan/CNVplex result, confirming the presence of the HKαα/αα variant. As for the CNVplex assay, we identified a large fragment deletion mutation (Chr16:50359-180299del; 129 kb; heterozygote) (Figure 2, Patient 849) and a large fragment duplication mutation (Chr16:113125-183148dup; 70 kb; heterozygote) (Figure 2, Patient 440), both of which were confirmed by genomic quantitative PCR (qPCR) (Figures 2B,D).

**TABLE 4:** Different results between SNPscan/CNVplex assay and routine PCR.

Sample ID	Age/Sex	MCV,fl	MCH,pg	Hb, g/L	HbA2, %	Results of routine PCR	Results of SNPscan and CNVplex assay
440	26 years/F	78.8	27.5	131	3	NA	Chr16:113125-183148dup
849	33 years/F	63	20.1	115	2.3	NA	Chr16:50359-180299del
312	35 years/F	88.9	30.2	96	2.3	-α^3.7^/αα	HKαα/αα
760	28 years/F	81.3	25.3	133	2.4	--^SEA^/αα	--^SEA^/ααα^anti4.2^
391	35 years/F	93.3	31.5	127	2.4	NA	ααα^anti3.7^/αα
468	31 years/F	89.3	28	128	2.3	NA	ααα^anti3.7^/αα
621	36 years/F	65.6	19.8	83	2.1	NA	ααα^anti3.7^/αα
672	23 years/F	72.5	23.9	105	2.4	NA	ααα^anti3.7^/αα
728	25 years/F	88	29.2	132	1.5	NA	ααα^anti3.7^/αα
737	24 years/F	83.7	28.9	144	1.6	NA	ααα^anti3.7^/αα
767	30 years/F	79.1	25.8	117	2.4	NA	ααα^anti3.7^/αα
808	34 years/F	85	26.5	93	2.4	NA	ααα^anti3.7^/αα
369	36 years/F	65.4	20.6	97	5.5	*HBB*:c.126_129delCTTT	ααα^anti3.7^/αα; *HBB*:c.126_129delCTTT Hete
739	42 years/F	66.5	21.22	95	5	*HBB*:c.126_129delCTTT	ααα^anti3.7^/αα; *HBB*:c.126_129delCTTT Hete
118	27 years/M	78.9	25.5	155	1.7	-α^4.2^/αα	-α^4.2^/αα; *HBA1*:c.223G>C Hete
95	36 years/F	91.2	30.1	123	2.4	NA	ααα^anti4.2^/αα
158	29 years/F	77.7	24.3	103	2.2	NA	ααα^anti4.2^/αα
190	28 years/F	65.7	20.8	106	4.8	*HBB*:c.316-197C>T	ααα^anti4.2^/αα; *HBB*:c.316-197C>T Hete
34	32 years/F	90.6	28.5	158	2.7	NA	*HBB*:c.-10_-7delAAAC Hete

## 4 Discussion

Thalassemia is one of the most common genetic diseases in the world, effective genetic testing is crucial for preventing thalassemia. In this study, we used SNPscan combined with CNVplex assay to detected 76 types of mutations of *HBA1/HBA2* and *HBB* among a total of 1,026 Chinese with abnormalities of blood testing. Mutations in *HBA1/HBA2* or *HBB* were found in 302 samples, with overall diagnostic rate of 29.43%. The most common allelic mutation of α-thalassemia was--^SEA^, while the most common allelic mutations of β-thalassemia were IVS-II-654 and CD41-42. The mutation spectrum in this research is similar to previous report in southern China (Shang et al., 2017). We also identified several rare α-globin gene homologous recombination types and large structural variations, such as ααα^anti3.7^, HKαα, and large segmental duplications or deletions. These structural variants are often difficult to detect by traditional methods, making them prone to misdiagnosis or omission in clinical diagnosis (Liu et al., 2000). To evaluate the value and application potential, the comparison of SNPscan/CNVplex assay and routine PCR of samples with positive results was also carried out. Among the 1,026 subjects, 19 (1.85%) had discordant results between SNPscan/CNVplex assay and PCR. Sanger sequencing and specially designed PCR verified the accuracy of the SNPscan/CNVplex assay and showed an increase in detection of 6.71%**.** SNPscan or CNVplex assay has been employed in the molecular diagnosis of other genetic disorders, such as expanded carrier screening, congenital deafness and bleeding disorder (Hu et al., 2022; Liang et al., 2017; Zhang et al., 2015). This combined strategy has been employed in the diagnosis of thalassemia. Wei *et al* evaluated SNPscan/CNVplex assay, which covers 67 thalassemia loci, and found that it achieved 100% accuracy (Wei et al., 2024). In our study, we expanded the number of testing sites from 67 to 75 and carried out parallel studies on a large cohort, through which a number of rare SNVs, indels and CNVs were identified. Our study provides further evidence supporting the clinical application of SNPscan/CNVplex assay; this technology is a method superior to routine PCR.

In this study, routine PCR assay missed triplications (including ααα^anti3.7^ and ααα^anti4.2^) in 14 subjects (of 1,026; 1.36%). The incidence of a-globin triplications was as high as 1%–2% among the general population in southern China (Xi et al., 2023) Non-allelic homologous recombination between the *HBA1* and *HBA2* genes results in an increased copy number of α-globin genes, such as ααα/α or ααα/αα (Motiani et al., 2024). Although individuals carrying the “ααα” genotype alone generally will not present with significant clinical symptoms, the co-existence of this genotype with β-thalassemia mutations (such as β^0^/β^+^ or β^0^/β^0^) can exacerbate the imbalance between α- and β-globin chains, eventually leading to moderate to severe thalassemic phenotypes (Sundaresan et al., 2023; Trehan et al., 2001). In this study, three patients were found to carry both ααα^anti3.7^ or ααα^anti4.2^ with β-thalassemia-associated mutations, presenting with microcytic hypodermic anemia (Table 4, Patient 190, 369 and 739). However, since most carriers exhibit no significant abnormalities in routine hematological tests, the identification of α-globin gene triplications during preconception and early pregnancy genetic screening is particularly critical (Wang et al., 2020). Conventional methods such as Gap-PCR or reverse dot blot hybridization primarily target common deletion mutations and are generally inadequate for detecting recombination or multiple-copy variants (Ge et al., 2024). As these rearrangements fall outside the range of traditional assays, carriers of homologous recombination types may go undetected, hindering accurate risk evaluation for offspring (Zhang et al., 2025).

Furthermore, we used CNVplex assay identified one large fragment deletion (Patient 849) and duplication (Patient 440) mutation, the two large fragment CNVs contain entire *HBA*. Since this duplication does not include other dosage-sensitive genes, its predicted clinical effects are analogous to those of α-globin triplications; whose clinical manifestations only occur when compounded with β^0^ or β^+^ heterozygosity. Specifically, the patient carrying deletion had a MCV of 63.0 fL (reference range: 76.0-88.0 fL) and a MCH of 20.1 pg (reference range: 24.0-30.0 pg), indicating microcytic hypochromic anemia. We designed primers at the two ends of the deletion fragment and confirmed the deletion by qPCR. In comparison to the common types of α-globin gene deletions observed in clinical settings, the deletion fragment in this case is larger and includes genes such as *HBZ*, *HBM*, *HBA1*, and *HBA2*. As for the high-throughput sequencing applied in thalassemia screening, NGS and TGS exhibit clear advantages in the simultaneous genotyping of SNVs and indels. However, detection of large rearrangements in CNVs and α-globin gene triplications from NGS data is challenging owing to the highly homologous *HBA* genes and the technology’s natural limitations such as short read lengths (limited by a read length of 300 bp), GC-content bias, panel design (Teo et al., 2012). As for TGS, it currently possesses significant technical advantages in diagnosing thalassemia, the longer reads nature of the TGS permits haplotype-phasing that is superior for variant discovery on the homologous genes and CNV calling. Although the high cost and the lengthy turnaround time for tests limit its widespread application in screening and diagnosing, larger fragment deletions exceeding 80 kb may remain undetected without customized TGS testing (Hassan et al., 2023). The CNVplex high-throughput linkage-dependent probe amplification technology is used to accurately detect deletion or duplication mutations in each exon of a target gene or any deletion or duplication mutation in any non-duplicated target segment greater than 60 bp. CNVplex, a copy number variation detection technique with high sensitivity to dosage changes, proved effective in identifying large fragment deletion and duplication in this study, demonstrating superior accuracy compared to traditional methods. Therefore, incorporating these mutations into routine screening panels and applying appropriate technologies for precise detection can improve both the coverage and effectiveness of thalassemia prevention strategies. This is particularly relevant in high-prevalence regions such as southern China (Lai et al., 2022).

Nevertheless, this study focused on 76 known hotspot mutations associated with thalassemia. Although the CNVplex technique can indicate potential CNVs, novel variants or CNVs less than 200 bp beyond the detection range may not be fully captured. For individuals presenting with clinical symptoms but lacking detectable mutations in SNPscan/CNVplex assay, NGS or TGS may be considered to improve diagnostic accuracy for rare SNVs and indels, while TGS may be a good choice for analyzing CNVs. In addition, the sample cohort in this study was exclusively derived from the Hunan region, which may introduce geographical bias. People carrying thalassemia variants are concentrated in southern China; combined with the large population, this results in a degree of bias in the results. Therefore, larger-scale studies with more diverse populations are needed to further refine the mutation spectrum of thalassemia. Clinically, accurate identification of carrier’s status is essential for improving birth outcomes and preventing congenital disorders. Genetic screening for thalassemia can be applied in premarital and preconception settings, as well as in prenatal diagnosis, and plays a critical role especially in high-risk combinations such as α^0^-thalassemia and β^0^-thalassemia (Esmaeilzadeh et al., 2022; Hossain et al., 2024). If more sensitive and high-detection-rate screening strategies can be widely implemented at the primary healthcare level, it would facilitate the early identification of high-risk couples, enable timely genetic counseling and medical intervention, and ultimately reduce the birth rate of children with severe forms of thalassemia.

## 5 Conclusion

The combined use of SNPscan and CNVplex technologies offers an effective strategy for the genetic screening of thalassemia. SNPscan is well-suited for parallel analysis of multiple SNP locis, while CNVplex enables detection of CNVs within the α- and β-globin gene clusters, particularly excelling in the identification of homologous recombination events and large fragment deletions. Our findings indicate that the combination of these two methods provides broader coverage of clinically relevant mutations and is superior to routine PCR.

## Data Availability

The original contributions presented in the study are included in the article/Supplementary Material, further inquiries can be directed to the corresponding authors.
