# Supplementary figures and images for: A comparative study of SNPscan/CNVplex assay and routine PCR in genetic analysis of thalassemia
Source: Front Genet. 2026 Jun 26;17:1819795. doi: 10.3389/fgene.2026.1819795 (PMC13349358; doi:10.3389/fgene.2026.1819795)

# Flowchart of CNVplex

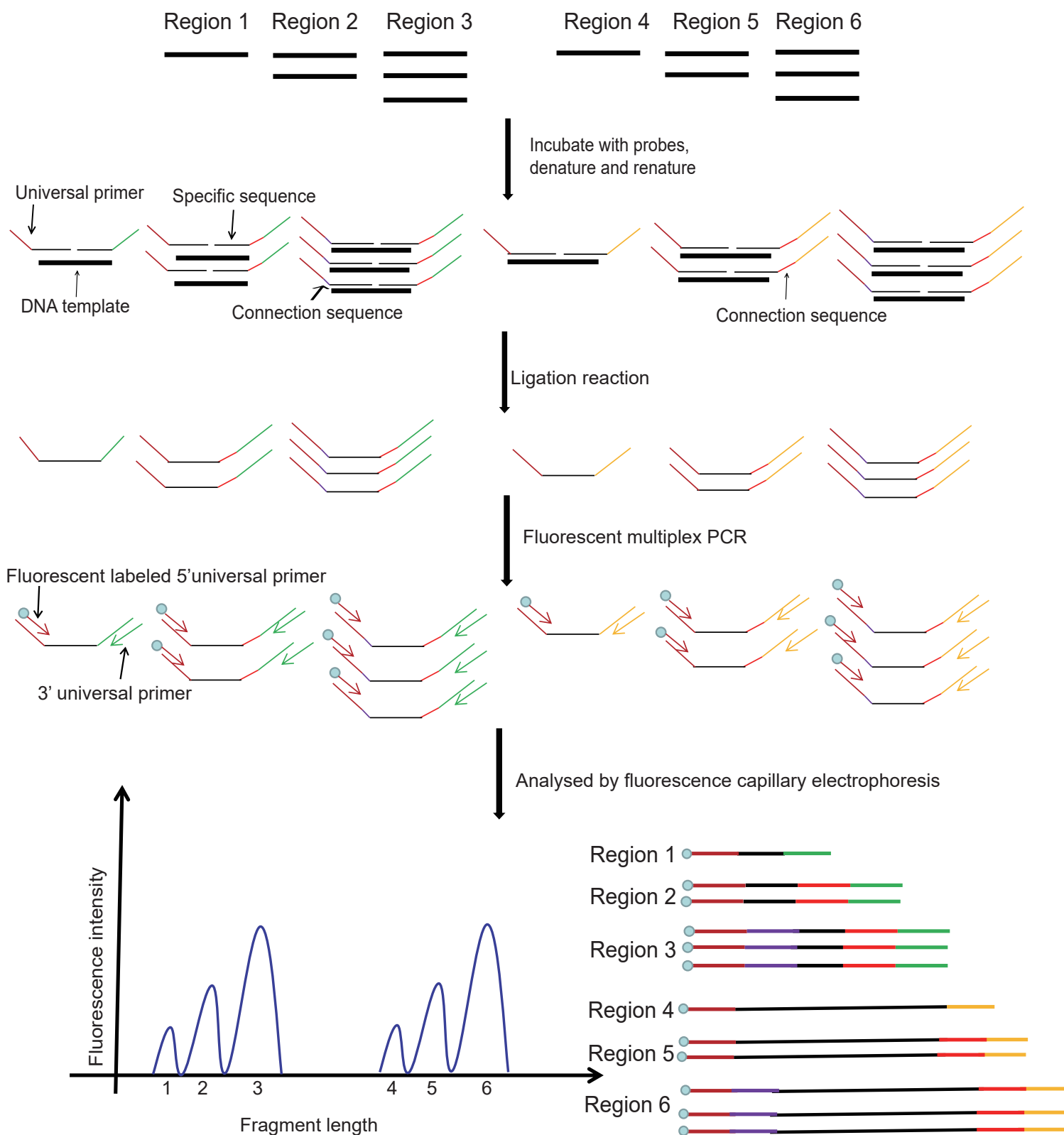

Supplement: Supplementary file 1 [file DataSheet2.pdf]

Flowchart of SNPscan

SNP1 C/T

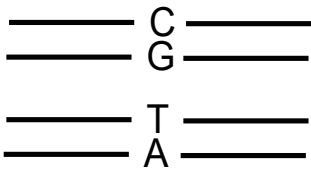

SNP2 G/C

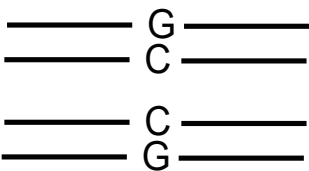

SNP3 G/A

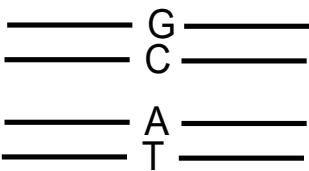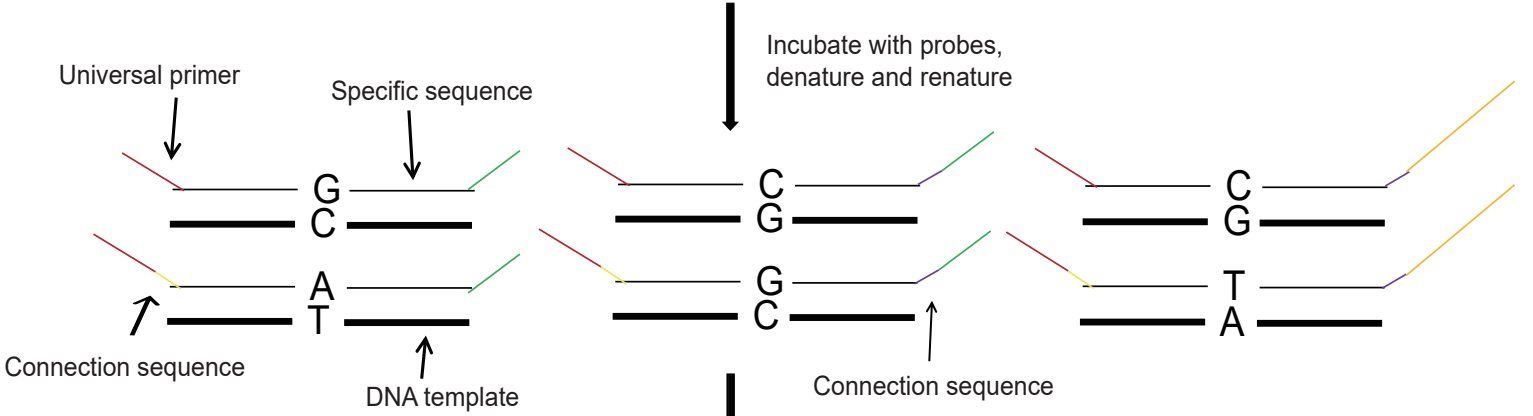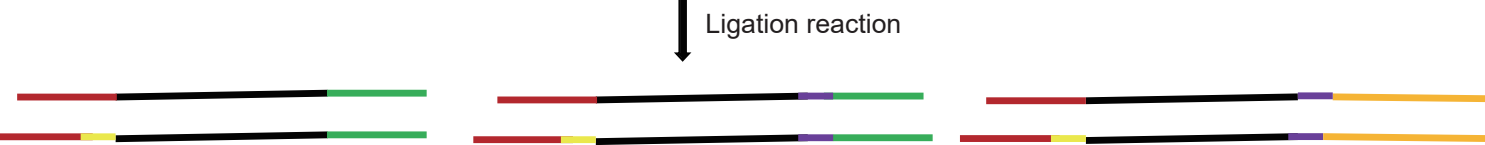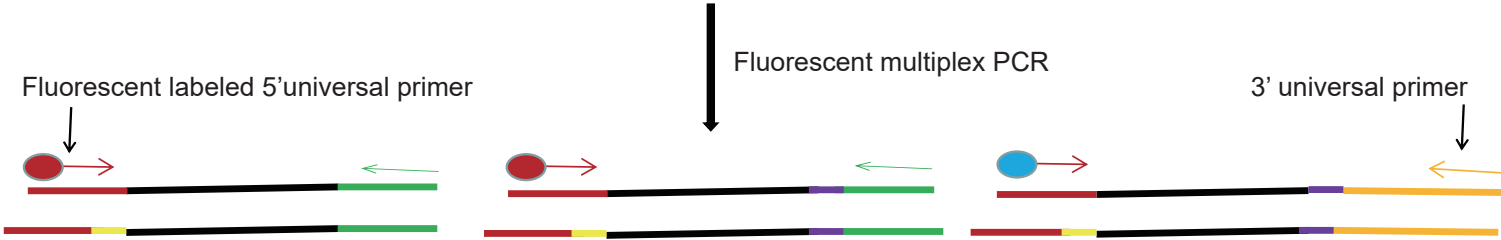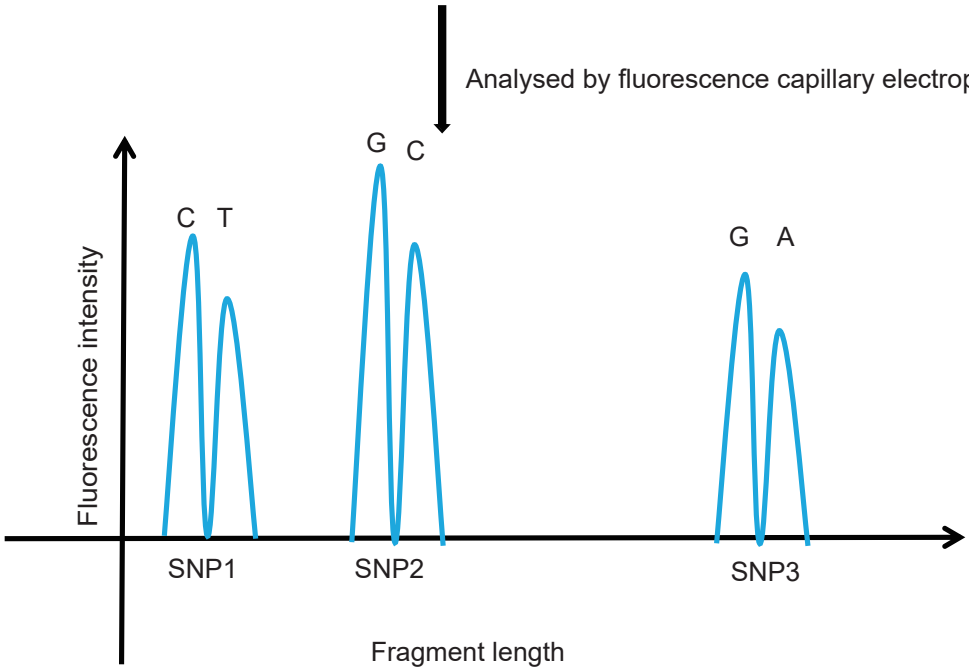

Supplement: Supplementary file 3 [file DataSheet1.pdf]
